# The process of stigma experience in the families of people living with bipolar disorder: a grounded theory study

**DOI:** 10.1186/s40359-022-00999-y

**Published:** 2022-11-29

**Authors:** Maryam Latifian, Ghoncheh Raheb, Riaz Uddin, Kianoush Abdi, Rosa Alikhani

**Affiliations:** 1 Department of Social Work, University of Social Welfare and Rehabilitation Sciences, Tehran, Iran; 2 Psychosis Research Center, University of Social Welfare and Rehabilitation Sciences, Tehran, Iran; 3 Institute for Physical Activity and Nutrition, School of Exercise and Nutrition Sciences, Deakin University, Melbourne, Australia; 4 Department of Rehabilitation Management, University of Social Welfare and Rehabilitation Sciences, Tehran, Iran; 5 Psychosis Research Center, University of Social Welfare and Rehabilitation Sciences, Tehran, Iran

**Keywords:** Stigma, Bipolar disorder, People living with bipolar disorder, Qualitative research, Grounded theory

## Abstract

**Background:**

One of the most challenging issues faced by families of people living with bipolar disorder is stigma. This study was conducted to explain the process of stigma experience in the families of people living with bipolar disorder using the grounded theory method.

**Methods:**

Data for this study were collected through semi-structured interviews with participants in Razi Psychiatric Hospital in Tehran, Iran, via purposive sampling and field notetaking. The dependability, conformability, and transferability measures were included to support the data accuracy and robustness, and MAXQDA 2020 software was used to facilitate data coding. The Strauss–Corbin method was used to analyse the data.

**Results:**

A total of 20 family members of people living with bipolar disorder, four people living with bipolar disorder, and three mental health professionals participated in this study. The analysis of participants’ experiences led to identifying 64 subcategories, 21 categories, and six main concepts, including social deprivation, being labelled, cultural deficiency and lack of awareness, economic challenges, forced acceptance of the existing situation, and social isolation.

**Conclusion:**

Families of people living with bipolar disorder experience social deprivation, social isolation, and social rejection, which have irreparable consequences for them. Overcoming stigma in these families should be a priority of policymakers and planners in the field of psychosocial health.

## Introduction

Stigma refers to prejudiced attitudes, stereotypes, and discriminatory behaviours embedded in a biased social structure against a specific group. The particular group refers to individuals distinguished from other members of society due to a social, psychological, or physical label [1, 2]. Psychiatric patients are one of these groups in such a way that stigma in patients with mental illnesses and their exclusion from the community is as old as the history of humanity [3, 4].

The prevalence of type 1 bipolar disorder in the general population is 1.5–2.1% and in the intensive care unit (ICU) is 21–26% [5–8]. The aggregate lifetime prevalence of all types of bipolar disorder was reported to be 5% [9]. Experts define bipolar disorder via episodes of mania, depression, or mixed psychotic or non-psychotic states. People living with bipolar disorder experience multiple mood swings, and the unpredictability of this disorder confuses patients and their families [10, 11].

Stigma is an essential issue in patients with bipolar disorder and their families because the consequences of stigma in this group of diseases are more than the disease itself [12, 13]. Individuals who are socially perceived as the family and close associates of a stigmatised person are also affected by the consequences of stigma, which Goffman calls “sympathetic others.” Thus, stigma is transmitted from people living with bipolar disorder to their family through communication, and Goffman calls this general perception of communication as “contagious stigma” [14, 15].The family members of people living with bipolar disorder experience internalised stigma because of being discriminated against and labelled by others. Internalised stigma, or self-stigma, refers to the process through which an individual becomes aware of people’s negative attitudes towards themselves and finally admits them. Internalised stigma refers to a type of identity transformation during which a person loses their previous (or expected) identity and adjusts themselves to the disgraceful views of people [16–18].

According to studies, more than 60% of people living with bipolar disorder return to their original family after discharge from psychiatric centres [19, 20]. Therefore, the family is the first and most important source for caring for these patients. Some changes occur in the families of people living with bipolar disorder that cause other family members to be unable to reach their maximum potential in different areas. Stigma experienced in these families can have adverse consequences, such as feelings of shame and frustration, distance from others, and being exposed to injustice and discrimination [21, 22]. Numerous studies have shown that 40% of families hide their patients’ hospitalization from others, and the fear of being stigmatised is considered the most critical and significant obstacle for patients and their families to benefit from medical services and social support [23–25].

Although culture is distinct from stigma, specific beliefs and interpretations of one’s culture may lead to stigma. Stigma is known as a universal phenomenon. However, its experience and the discrimination imposed in various countries are different. The stigma of mental illness may is rooted in culture and influenced by historical, social, and cultural factors specific to each community and makes sense to different degrees among people of other societies [26, 27].

According to Becker’s theory, labelling is strongly influenced by the social characteristics of the labeler, the person being labelled, and the social position in which the interaction takes place [28]. Most studies on the concept and dimensions of stigma are from developed countries. Currently, there is a lack of studies in low-and middle-income countries, although a significant percentage of psychiatric patients live in these countries [20].

Governments from LMICs^1^ spend the lowest percentages on mental health worldwide. The World Health Organization (WHO) has reported that the treatment gap for serious mental disorders is 35–50% in developed countries and 76–90% in LMICs [29, 30]. Iran is a low-or middle-income country with a high rate of bipolar disorder, with an estimated prevalence of bipolar disorder type 1 in Iran to be 2–4% [31]. The experience of stigma and discrimination is common in this patient group; however, research in this area is minimal and insufficient.

There is a culture of collectivism in Iran that emphasises collectivist norms and interdependent self-concept; as a result, family members of people living with bipolar disorder are more concerned with how others view the family than with the negative impact of the disorder on themselves [32, 33]. To date, in Iran, no study has comprehensively investigated the process of stigma formation and experience in the families of people living with bipolar disorder. Therefore, given the insufficiency of existing studies, the increasing consequences of social stigma on this vulnerable group, and the cultural dependence of this phenomenon, more attention to the issue of stigma in the families of people living with bipolar disorder seems necessary. The present aimed to explain the process of stigma experience in the families of people living with bipolar disorder.

## Method

### Design and participants

This qualitative research was conducted following the grounded theory method in the first half of 2021 at Razi Psychiatric Hospital in Tehran, Iran. People living with bipolar disorder, family members of bipolar disorder patients, and mental health professionals participated in this study.

The primary sample of this study was family members of people living with bipolar disorder. Interviews with other groups were performed to increase the richness of the study. Initially, semi-structured interviews were conducted with 17 family members of people living with bipolar disorder who were selected by targeted sampling method. Then, theoretical sampling continued by interviewing three more family members of people living with bipolar disorder to fill the categories and expand the theory until data saturation was achieved.

However, sampling continued to increase the richness of the study and the validity of the data. Further, four people living with bipolar disorder and three mental health professionals were interviewed.

Inclusion criteria for the main participants of the study, i.e., the families of people living with bipolar disorder, including currently residing in the same house with the patient, having lived in the same house with the patient for at least two consecutive years, and having passed at least two years since a family member developed bipolar disorder. The cut-off of two years was based on an expert panel of psychiatrists and social workers at Tehran, who noted that at least two years should pass from the onset of symptoms of bipolar disorder. The time is crucial so that family members can gain enough experience living with a bipolar patient to talk about the consequences of stigma. Only one family member was allowed to participate in the study. If a patient participated, their family members were ineligible to participate; similarly, in case a member of the family of a patient participated, the patient was ineligible.

Inclusion criteria for people living with bipolar disorder were: to be diagnosed as a bipolar patient by a clinical examination of a psychiatrist, to have a history of hospitalisation in a psychiatric hospital, to have sufficient knowledge and insight to answer questions at the time of the interview and to be willing to participate and speak in research.

Additionally, inclusion criteria for mental health professionals were: has studied in one of the fields of social work, psychiatry or psychology, is employed in a psychiatric hospital, has at least five years of experience in the field of psychiatry and is willing to participate in research.

### Data collection

Data were collected using semi-structured interviews, first asking an open question and then follow-up and probing questions selected based on participants’ responses and field notetaking. Given the fundamental nature of qualitative research, the decision regarding the best data collection methods and from whom and how to collect data was finally made in the field of study and while conducting it.

The interview questions were open-ended and non-judgmental. The interview usually began with the questions: "Talk about your living conditions with a bipolar patient at home, or how would you describe living with a bipolar patient in general?" Subsequent questions from the participants were based on their answers. The next questions were selected by the interviewer to get more details of the topic under discussion. For example, the participant was asked to explain further or give examples of the issues raised. After each interview, the analysis was performed by the research team, and then the following interview was conducted based on the information obtained.

Each interview lasted 30–90 min. According to prior coordination and permission from the participants, all interviews were recorded by a digital tape recorder and were then written and transcribed verbatim.

### Data analysis

Sampling and data analysis lasted eight months (April 2021 to November 2021). The researcher read each recorded interview and its related notes immediately and line by line during the first few hours and noted the concepts that emerged from the interviews. To facilitate the process of coding and analysing data, MAXQDA2020 software was used, and a codebook was generated. The consensus of each code was reached after discussion and exchange of views between members of the research team.

According to the Strauss–Corbin model, data coding was performed in three stages [34]: Open, axial, and selective. In open coding, semantic units were identified, and then phrases and sentences of a similar nature were merged to form subcategories. The subcategories formed in the previous step were classified in axial coding to create inclusive and exclusive categories. All the steps were considered simultaneously in the selected coding, and the central concept was identified; then, the following interview was performed, and the above steps were repeated. The back-and-forth process between data and their analysis was performed simultaneously with data collection and continued until theoretical saturation. We presented an example of the formation of concepts in Table 1.

**Table 1:** An example of the formation of concepts

Concept	Categories	Sub-categories	A sample of semantic unit
Forced acceptance of the existing situation	Indifference and disregard for the existing situation	Ignorance of the looks of others	In the beginning, I was very upset by the words of people, but I’ve decided to be carefree. Should I ruminate constantly? Should I commit suicide? What should I do when people are talking? I told my daughter, ‘we shouldn’t care about these issues, and we should make our lives’
		Not arguing against inappropriate words of others	
		Entertaining themselves, not to think of the difficult conditions of life	
		Ignorance of patient’s behaviours	
	Identifying families with similar problems	Comparing their situation to families with disabled members	It was God’s will that my life is like this. There’s a family with a disabled individual; well, it’s our destiny to have it. People should understand that this is a disease like any other disease and do not rub salt into our wounds
		Comparing their situation to families with drug user members	
	Silence and surrender	Trying to normalise the situation	I don’t like others to know more or less about my life and my sorrow and grief. That’s why I put everything in my heart and don’t talk to anyone about my problems
		Keeping sadness and grief with you	
		Silence in the face of immoral suggestions	
		Surrender to circumstances	
	Distancing from others	Multiple changes of place of residence	It has happened many times that I changed my place of residence. When they don’t know about it, they behave with a lot of dignity and respect, but when they understand, everything changes very easily; as I was humiliated among the relatives, so it is among the neighbours
		Limiting communication with others	
		Reducing the amount of leaving the house	
	Hiding the disease	Not talking to others regarding the disease of the affected family member	Everyone who understands that she’s my sister starts to give advice… That’s why we try our best not to let anyone know that our sister’s sick …
		Imprisoning the people living with bipolar disorder at home	

At the end of the data collection stage, two FGD^2^ sessions were conducted. These meetings were conducted to check the data's validity, and the data obtained from FGD sessions were not included in the analysis. By comparing the information obtained from the FGD sessions with the main data that obtained from interviews that were included in the analysis, we concluded that there is consistent between them.

Five mental health specialists attended one of these meetings, including two social workers, one psychologist and two psychiatrists. Another meeting was held in one of the hospital rooms with the presence of four family members of bipolar patients. The participants in the FGD sessions had not participated in the interviews before.

A trained research student with expertise in qualitative research, including one-to-one interviews and experience in psychiatric patient care, conducted the interviews and facilitated focus group discussion sessions.

### Data robustness

The selected sample represented different age groups, educational status, illness duration, and the number of hospitalisations of the patients. To evaluate data reliability, the interview texts were studied and coded again by the researcher after a few days, and the results were compared with the previous coding. Also, the research colleagues and two observers outside the research team who were familiar with the qualitative research method evaluated parts of the tapes and texts of the interviews along with the extracted codes, subcategories, and categories.

### Ethical considerations

The study was approved by the University of Social Welfare and Rehabilitation Sciences ethics review committee (Ref: IR.USWR.REC.1399.249). Informed consent was obtained from the parents or legal guardians of each of the patients. Other participants provided their informed consent to participate in the study.

## Results

Twenty-seven individuals, including 20 family members of people living with bipolar disorder, four people living with bipolar disorder, and three psychiatrists participated. We summarised the characteristics of study participants in Table 2.

**Table 2:** Characteristics of research participants

Sampling	Relationship with patient	Age	Education	Occupation	Duration of illness (year)	Number of hospitalizations since diagnosis
*Bipolar patient’s family members*						
Targeted	Sister	48	Diploma	Cleaner	8	3
Targeted	Spouse	50	Middle school	Carpenter	13	4
Targeted	Spouse	53	Associate	Employee	9	2
Targeted	Child	59	Bachelor	Employee	6	3
Targeted	Brother	49	Bachelor	Self-employment	6	2
Targeted	Mother	65	Diploma	Housewife	30	4
Targeted	Brother	42	Diploma	Self-employment	7	2
Targeted	Mother	50	Illiterate	Cleaner	9	5
Targeted	Spouse	54	Associate	Retired	12	4
Targeted	Father	51	Illiterate	Factory worker	15	6
Targeted	Brother	32	Associate	Self-employment	9	5
Targeted	Sister	47	Bachelor	Nurse	16	7
Targeted	Mother	69	Elementary school	Cleaner	13	2
Targeted	Spouse	47	Illiterate	Vendor	20	4
Targeted	Mother	66	Illiterate	Housewife	14	7
Targeted	Father	68	Middle school	Construction worker	20	8
Targeted	Sister	29	Bachelor	Housewife	9	5
Theoretical	Sister	38	Bachelor	Elderly nurse	11	5
Theoretical	Brother	40	Middle school	Self-employment	14	3
Theoretical	Father	61	Illiterate	Construction worker	18	5

Sampling	Age	Gender	Marital status	Education	Occupation	Illness duration (year)	Frequency of hospitalizations
*People living with bipolar disorder*							
Theoretical	39	Male	Single	Illiterate	Unemployed	13	8
Theoretical	52	Male	Married	Illiterate	Unemployed	20	3
Theoretical	47	Male	Divorced	Illiterate	Janitor	12	3
Theoretical	34	Female	Divorcee	Illiterate	Unemployed	10	6

Sampling	Age	Gender	Expertise	Education	Duration of activity in the field of psychiatry (year)
*Mental health professionals*					
Theoretical	43	Female	Social worker	Master	11
Theoretical	51	Male	Social worker	Master	14
Theoretical	49	Female	Psychiatrist	Doctorate	9

The main concepts extracted in this research included social deprivation, labelling, cultural deficiency and lack of awareness, economic challenges, forced acceptance of the existing situation, and social isolation. The following is a description of each of these concepts.

**Fig. 1:**
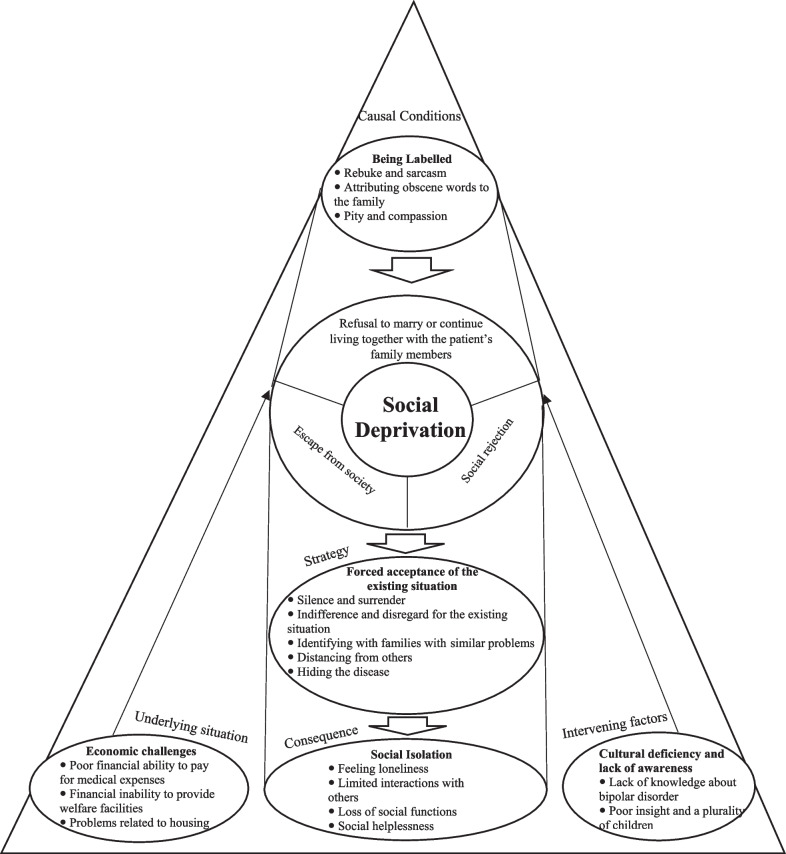
The “social deprivation” model regarding the stigma phenomenon in the families of people living with bipolar disorder

Figure 1 illustrates the “social deprivation” model regarding the stigma phenomenon in the families of people living with bipolar disorder.

### Social deprivation

According to Strauss and Corbin, the central phenomenon is a category that has the power of analysis and is related to other categories [34]. The central phenomenon is the main theme of the research. The concept of “social deprivation” is created following the labelling of family members of bipolar patients by those around them and the community, and family members see different actions and reactions in dealing with it. This variable and its sub-categories were frequently heard in the participants' speeches. After paying close attention to the text of the data, its relationship with other categories was determined. So the concept of “social deprivation” was considered the central phenomenon due to the most explanatory association with different categories. This concept contains three categories, referred to in the following in some participants’ interviews by these categories.

- A) Social rejection: A patient’s 54-year-old spouse:
  “When the locals see us, it’s as if they see something terrible and run away from us. My daughter says that everyone looks at her the same way in the park or school, and when she goes to play with them, they walk away. She comes home crying and says, why do the children distance themselves from me?”
- B) Refusal to marry or continue living together with the patient’s family members: A patient’s 59-year-old child:
  “One of my brothers has a doctorate in economics, and he’s a very good boy in every aspect, but so far he has gone to several places to propose marriage, and as soon as they find out that our mother is ill, they immediately reject him. Why shouldn’t my brother have a good marriage with this situation?”
- C) Escape from society: A patient’s 38-year-old sister:
  “Two of my cousins got married, and our aunt didn’t invite us at all. We try not to be in public. We even sent our sister on a honeymoon and didn’t allow her to have a wedding because no one came to her wedding, and we didn’t want anyone to come as well.”

### Being labelled (causal conditions)

Based on the findings of this study, in response to a research question about how families of bipolar patients experience the stigma process, this process takes place in a context in which the family members of bipolar patients, on the one hand, they are persecuted a lot by the people living with bipolar disorder. On the other hand, due to inappropriate and unusual words and behaviours of the patient in the residence and the harassment of those around them, they experience shame and embarrassment. Often, the reactions of those around them to these families cause them to be labelled. Thus, Categories such as attributing obscene words to the family, rebuke and sarcasm, and pity and compassion under a single concept called “labelling” were known as causal conditions due to their direct impact on the central phenomenon. The following are some of the participants’ interviews in this regard.

- A) Attributing obscene words to the family: A patient’s 51-year-old father:
  “If you want to get a wife for your child, people say they shouldn’t give him a wife; one of their children is in the mental hospital. I heard with my own ears that they say ‘they are all crazy’.”
- B) Rebuke and sarcasm: A patient’s 65-year-old mother:
  “In the family, I heard sarcasm from my sister; she mocked my child. They didn’t allow my son to approach their parrot cage. They said ‘all your family has jinn in you.'"
- J) Pity and compassion: A patient’s 47-year-old wife:
  “Relatives say: ‘Oh! What a pity that you became this man’s wife. What a pity for your beauty. Divorce him sooner until you are young. What is this life that you have?! You’re going to be crazy like him'.”

### Cultural deficiency and lack of awareness (intervening factors)

Labelling family members of bipolar patients is far more common and worse in a society where people are culturally deficient and unaware. Because in these societies, ignorance of the family and others about the nature of bipolar disorder, lack of information about how to treat the patient, misconceptions about hospitalisation to a psychiatric hospital and lack of information about the innocence of the family makes family members easily labelled and ridiculed and insulted by others. Cultural deficits in the local community of these families can be due to the low education of the family and others, the growth of people in large families who have not had enough time to care for their children, lack of familiarity with parenting skills and patriarchal culture (In which the continuation of education or employment of girls is opposed and the only purpose of a girl's life is marriage and girls in such a culture are humiliated and insulted from the beginning of childhood). Thus, the categories of lack of awareness about bipolar disorder, poor insight and a plurality of children, and misogynistic thoughts were identified under a single concept called “cultural deficiency and lack of awareness” as intervening factors affecting stigma coping strategies. The following are some interviews in this regard.

- A) Lack of awareness about bipolar disorder: A patient’s 32-year-old brother:
  “Maybe one in every 100 people knows that this is also a disease and should be tolerated, but the rest don’t know anything. We ourselves also don’t know much about it. We don’t know at all what to say to him and what not to say. Sometimes he’s distraught to hear the news, and sometimes he treats normally ... This is what confuses us.”
- B) Poor insight and a plurality of children: A patient’s 29-year-old sister:
  “Most of our relatives are illiterate. Their culture is poor, and everyone’s used to wandering and interfering in each other’s life. Our parents, who had so many children, didn’t care about raising their children, and everyone bullied or even beat us.”
- C) Misogynistic thoughts: A patient’s 38-year-old sister:
  “I also liked to study and go to work, but my father had an idea that if my daughter left Ardabil, she would have a problem. That’s why he limited us and didn’t let us study and work.”

### Economic challenges (contextual conditions)

The family's financial inability is considered a context for forming stigma. Because in low-income families, people do not have the financial means to provide the patient's medication and pay for the hospital, which causes the treatment to be abandoned and the patient's symptoms to worsen. Following the exacerbation of the symptoms of the disease, the stigma experienced in families increases. In this study, financial inability to provide welfare facilities, poor financial ability to pay for medical expenses, and housing problems were identified as “economic challenges”, a basis and context for stigma formation. The following are some interviews related to this concept.

- A) Financial inability to provide welfare facilities: A patient’s 50-year-old wife:
  “If you come and see, you’ll understand. We have nothing at home; facilities zero ... we don’t even have water, electricity, and gas.”
- B) Poor financial ability to pay for medical expenses: A patient’s 61-year-old father:
  “Now, I’m worried about how I can pay for my patient’s discharge expenses from the psychiatric hospital?”
- C) Housing problems: A patient’s 50-year-old mother:
  “Our house is 60 meters and very small because we are five people. At present, my husband pays half of his income for installment, and we aren’t in a good financial status to go and get a house somewhere else.”

### Forced acceptance of the existing situation (strategy)

The reactions of family members of bipolar patients to stigma and the resulting Social Deprivation vary. Categories of indifference and disregard for the existing situation, identifying with families with similar problems, silence, and surrender, distancing from others, and hiding the disease, were recognised as “forced acceptance of the existing situation”, as specific actions or interactions resulting from the central phenomena. The following are some participants’ interviews in this regard.

- A) Indifference and disregard for the existing situation: A patient’s 68-year-old father:
  “Sometimes I play Shirazi music to be entertained and to forget the problems of life; now, I've been carefree for several years; otherwise, I'd have been ruined.”
- B) Identifying with families with similar problems: A patient’s 69-year-old mother:
  “I always say to myself that there are many unfortunate mothers like me. This child is God’s creature… One is paralyzed, one has no legs, one has no arms; this is also the case. My destiny has always been to be alone.”
- C) Silence and surrender: A patient’s 47-year-old sister:
  “One breaks hearing people’s words, I’ve to not to bat an eyelid; I can’t fight him or behave him like himself and say your son is also addicted ... I’ve to keep the sadness in my heart and do not say a word.”
- D) Distancing from others: A patient’s 66-year-old mother:
  “I’ve lost contact with my relatives. I don’t even have any contact with my daughter and son because I can’t tolerate the looks of my bride and groom. I’m not going anywhere, and I’m always at home. I even told my husband that it would be better to change our house and leave this neighbourhood.”
- E) Hiding the disease: A patient’s 61-year-old father:
  "We’ve got a pillar in the house. I tie him to the pillar with chains because he’ll dishonour us if he goes out of the house. My mother used to say that we shouldn’t take her to the hospital or that if we did, no one would understand because it’s very bad among relatives to say that they’ve taken and admitted him to a psychiatric hospital and that it’s better that no one understands.”

### Social isolation (consequence)

With increasing stress in these families, the possibility of family breakdown increases. Sleep disorders, self-harm, tendency to use drugs and the emergence of neurological diseases appear. Emotional arousals such as mental turmoil and remorse, constant and unceasing stress and worry and feelings of shame and humiliation intensify in these families. Therefore, The categories of the feeling of loneliness, social helplessness, limited interactions with others, and loss of social functions were recognized under a single concept called “social isolation” as the consequences and results of stigma in the families of people living with bipolar disorder. The following are some participants’ interviews regarding the dimensions of social isolation.

- A) The feeling of loneliness: A patient’s 29-year-old sister:
  “I feel very lonely. No one understands me and my situation. I’m afraid to get married. I always say, what will happen if my fiancé’s family finds out that I have a mentally ill brother?”
- B) Social helplessness: A patient’s 50-year-old mother:
  “No matter how hard I try in my life, it’s useless and won’t work. Everyone says you are to blame. What’s my fault I kept the baby? I was young and inexperienced, and I took custody of the child; otherwise, I could also have left and gone and been comfortable like any other woman. Even now, I can’t sleep at night because of worries about my future and that of my son.”
- C) Limited interactions with others: A patient’s 53-year-old wife:
  “We’re in a dead-end alley. We’ve six neighbors in total, and we’ve sulked with five of them because of the same word. I go shopping for days. I wait for the weather to get dark. At 8 pm, I go shopping so that I see no one in the alley.”
- D) Loss of social functions: A patient’s 42-year-old brother:
  “It’s happened to me a lot that when the employer finds out that my sister’s sick, he rejects me. If Zahra weren’t my sister, I wouldn’t have done construction work, and I could’ve had a better job.”

## Discussion

This study aimed to explain the process of stigma experience in the families of people living with bipolar disorder. According to the findings of this study, social deprivation, including social rejection, refusal to marry or continue to live together with the patient’s family members, and social isolation, were identified as the most critical concept in the stigma of the families of people living with bipolar disorder, which can integrate all the data of this study as a central phenomenon. The findings of our study are consistent with previous studies by Sadeghi [32], Reinares [35], and Anderson [36]. The rejection of family members of people living with bipolar disorder by society and the problems related to their education and employment have been raised as the most important concerns by previous researchers.

Items such as attributing obscene words to the family, rebuke and sarcasm, and pity and compassion, were identified as a broader concept called labelling as causal conditions affecting the creation of social deprivation. The results confirm the findings of Miklowitz [37], Goossens [38], and Sadeghi’s [32] who highlighted that being labelled of the families of people living with bipolar disorder’ as one of the most important underlying factors of stigma experience. Lack of awareness about bipolar disorder, poor insight and a plurality of children, and misogynistic thoughts were identified under a single concept called cultural deficiency and lack of awareness as intervening factors, which affect stigma coping strategies. This result is consistent with Bassirnia [3], Thome [39] and Ellison [2] studies. Cultural problems, traditional believes, lack of knowledge and awareness of the public in various fields, and insufficient knowledge and information about bipolar disorder have been introduced as facilitators of the process of stigma formation in the families of these patients.

In this study, financial inability to provide welfare facilities, poor financial ability to pay for medical expenses, and housing problems were identified under a concept called economic challenges as a basis for stigma formation. These findings are consistent with Grover [13] and Ellison’s [25] studies. These studies also emphasized the importance of financial and economic problems in stigma formation and believe. Previous studies noted that the phenomenon of stigma is experienced more by the vulnerable groups of society that cannot support themselves financially and have difficulties with the medical expenses associated with the treatment and management of bipolar disorders. The categories of indifference and disregard for the existing situation, identifying with families with similar problems, silence and surrender, distancing from others, and hiding the disease were recognised under a single concept called forced acceptance of the existing situation as specific stigma coping strategies. This finding supports the earlier findings of Richard [4] and Aziz [24]. They found that following stigma experience, the families of people living with bipolar disorder hide the disease from others, distance themselves from others as much as possible, and try to be indifferent to their difficult situation to reduce the disease complications so that they can have an easier life.

Finally, issues such as the feeling of loneliness, social helplessness, limited interactions with others, and loss of social functions were recognized under a single concept called social isolation as the consequences and results of stigma in the families of people living with bipolar disorder. These findings are consistent with the findings from the earlier studies by Shamsaei [7], Ganguly [26], Jonsson [27], and Grover’s [1]. These studies also reported social isolation and withdrawal from society as the most critical consequence of stigma in people living with bipolar disorder.

Although some of the results of the present study were consistent with the above studies, it should be noted that these studies have been conducted sparsely in different countries and have examined each part of the process of stigma experience in the family of bipolar patients and have often been done in quantitative research. So far, no study has been conducted in Iran to explain the process of stigma experience in the family of bipolar patients from beginning to end in a qualitative and in-depth and comprehensive manner.

The role of researchers' observations and their intentions and prejudices in the study results cannot be denied in general. However, we tried to minimize their impact on the research results. So that, in the process of interviewing and data analysis, the researcher was careful to leave aside the previous assumptions and any bias towards the data analysis and tried to report the events as they happened and heard from the participant's language and avoid mental interference.

More research, including using quantitative research methodology and more representative sample is needed to gain a more comprehensive understanding of stigma in the lives of people living with bipolar disorder and their family. The response of the participants may be limited by social desirability bias. As the participants were interviewed at the time of a clinical visit, their responses may have been influenced by the most recent visit at the hospital.

## Conclusion

The results of this research showed the process of stigma formation, coping strategies, and its consequences in the families of people living with bipolar disorder. In a society with multiple cultural complexities, the families of people living with bipolar disorder experience stigma following being labelled by the community members and are socially deprived in many aspects of their lives. Consequently, to return to normal life, they are forced to accept the situation and try to adopt a method of indifference and distance themselves from other people as much as possible to face people less. The result is that these individuals experience social isolation and exclusion and repeatedly experience the feeling of loneliness and social helplessness. In order to deal with the phenomenon of stigma and reduce its complications in the families of people living with bipolar disorder, it is necessary to do macro and micro planning separately for different stages. Therefore, it is suggested that, in the first place, programs to prevent the occurrence of stigma in the general public be developed to inform people and normalize bipolar disorder, and in the second place, specialized services be provided to the families of people living with bipolar disorder that are currently experiencing stigma and suffering from this condition.

## Data Availability

The data that support the findings of this study are available from the corresponding author upon reasonable request.

## Footnotes

1 Low- or middle- income country.

2 Focus group discussion.
